# Quality of life’ evaluation for individuals with enduring mental illness transitioning from institutional residential care to supported community living arrangements

**DOI:** 10.1192/j.eurpsy.2021.1881

**Published:** 2021-08-13

**Authors:** M. Usman, L. Spelman

**Affiliations:** 1 Markievicz House, Sligo Leitrim MHS, Sligo, Ireland; 2 Woodview Unit 9a, Galway Roscommon MHS, Galway, Ireland

**Keywords:** quality of life, Enduring, Rehabilitation, recovery

## Abstract

**Introduction:**

HSE ‘Vision for Change, 2006’ placed emphasis on person-centred, recovery-oriented, community-integrated treatment. The high support residential hostel in Tuam ‘Toghermore House’ is not integrated into the community and was scheduled to be closed as a residence in the last quarter of 2019. The individuals whom accessed support from Toghermore House were offered residential places in community settings in the urban area of Tuam with support plans according to individual assessed needs.

**Objectives:**

To assess the quality of life of individuals accessing the Rehabilitation & Recovery Service and who are residing in supported and independent accommodation in Tuam.

**Methods:**

Cross sectional study. Scales used were Manchester Short Assessment of Quality of Life Scale (MANSA) including both objective and subjective components and the INSPIRE scale which gathered information about the support and relationship each individual has with their assigned keyworker. SPSS 24 was used for data analysis.

**Results:**

27/32 responses. Mean age: 52 years, males: 78% and schizophrenia: most common primary diagnosis (52%), mean duration of illness: 28 years 3 months. 74% of individuals were satisfied with their life, 78% with their health and 56% with mental health. Only three individuals were employed and were happy with work and finances. 81.5% service users reported to have a good quality of life but only 59% felt in control of their lives.

**Conclusions:**

Majority of individuals reported having a good quality of life and being satisfied with their overall health and current living arrangements. Meaningful occupation and subjective supportive therapeutic relationships are predictors of enhanced quality of life.

**Disclosure:**

No significant relationships.

